# Experiences of Nursing Care among Older Adults Living with HIV

**DOI:** 10.1093/geroni/igaf122.2677

**Published:** 2025-12-31

**Authors:** Juh Shin, Yu-Ping Chang, Vanessa Cameron, David Vance

**Affiliations:** George Washington University, Ashburn, Virginia, United States; University at Buffalo, Buffalo, New York, United States; Vanessa Cameron Education & Consulting, Leavenworth, Washington, United States; University of Alabama at Birmingham, Birmingham, Alabama, United States

## Abstract

Despite advancements in HIV treatment, clinical care for people living with HIV (PLWH) remains suboptimal. To improve care delivery, it is essential to understand the lived experiences of PLWH individuals affected by HIV, particularly older PLWH adults who face unique challenges. This study aimed to explore perspectives regarding nursing care in older PLWH adults living with HIV and. Semi-structured individual interviews were conducted with 20 11 older adults living with HIVPLWH (50+). Thematic analysis was utilized to understand their experiences with nursing care. All interviews were recorded, transcribed, and systematically analyzed to identify key themes. Overall, participants described nursing care as a valuable source of support and generally high in quality. However, they also highlighted critical areas for improvement. Five key themes emerged: (1) Looking Beyond the Virus and T-Cells: the need for holistic care that addresses co-existing chronic conditions. (2) Identifying Emerging Care Needs: proactive assessments to detect evolving health concerns. (3) Unmet Psychological and Emotional Needs: addressing loneliness and social isolation. (4) Ensuring Accessibility and Culturally Appropriate Care: addressing barriers to healthcare access. (5) Supporting Patient Empowerment and Advocacy: enhancing education and resources for self-advocacy. While participants valued the nursing care they received, they emphasized the need for more comprehensive support, particularly in advocacy, social connection, care accessibility, and care coordination. These findings highlight critical gaps in current nursing practices and underscore the need for patient-centered, holistic approaches. Future research should focus on developing and implementing coordinated services to enhance the well-being of older adults with HIV.

